# Melatonin-Induced Water Stress Tolerance in Plants: Recent Advances

**DOI:** 10.3390/antiox9090809

**Published:** 2020-09-01

**Authors:** Mohamed Moustafa-Farag, Ahmed Mahmoud, Marino B. Arnao, Mohamed S. Sheteiwy, Mohamed Dafea, Mahmoud Soltan, Amr Elkelish, Mirza Hasanuzzaman, Shaoying Ai

**Affiliations:** 1 Institute of Agricultural Resources and Environment, Guangdong Academy of Agricultural Sciences, Guangzhou 510640, China; 2 Horticulture Research Institute, Agriculture Research Center, 9 Gmaa St, Giza 12619, Egypt; 11716103@zju.edu.cn (A.M.); mohameddafea@yahoo.com (M.D.); 3 Laboratory of Germplasm Innovation and Molecular Breeding, Institute of Vegetable Science, Zhejiang University, Hangzhou 310058, China; 4 Department of Plant Physiology, Faculty of Biology, University of Murcia, 30100 Murcia, Spain; marino@um.es; 5 Department of Agronomy, Faculty of Agriculture, Mansoura University, Mansoura 35516, Egypt; salahco_2010@mans.edu.eg; 6 Horticulture and Crop Science Department, Ohio Agricultural Research and Development Center, Columbus, The Ohio State University, Columbus, OH 43210, USA; dsoltan2012@gmail.com; 7 Vegetable Production under Modified Environment Department, Horticulture Research Institute, Agriculture Research Center, Cairo 11865, Egypt; 8 Botany Department, Faculty of Science, Suez Canal University, Ismailia 41522, Egypt; amr.elkelish@science.suez.edu.eg; 9 Department of Agronomy, Faculty of Agriculture, Sher-e-Bangla Agricultural University, Dhaka 1207, Bangladesh; mhzsauag@yahoo.com

**Keywords:** melatonin, water stress, drought, waterlogging, abiotic stress, antioxidants, stress signaling, phytohormones

## Abstract

Water stress (drought and waterlogging) is severe abiotic stress to plant growth and development. Melatonin, a bioactive plant hormone, has been widely tested in drought situations in diverse plant species, while few studies on the role of melatonin in waterlogging stress conditions have been published. In the current review, we analyze the biostimulatory functions of melatonin on plants under both drought and waterlogging stresses. Melatonin controls the levels of reactive oxygen and nitrogen species and positively changes the molecular defense to improve plant tolerance against water stress. Moreover, the crosstalk of melatonin and other phytohormones is a key element of plant survival under drought stress, while this relationship needs further investigation under waterlogging stress. In this review, we draw the complete story of water stress on both sides—drought and waterlogging—through discussing the previous critical studies under both conditions. Moreover, we suggest several research directions, especially for waterlogging, which remains a big and vague piece of the melatonin and water stress puzzle.

## 1. Introduction

**Figure 1:**
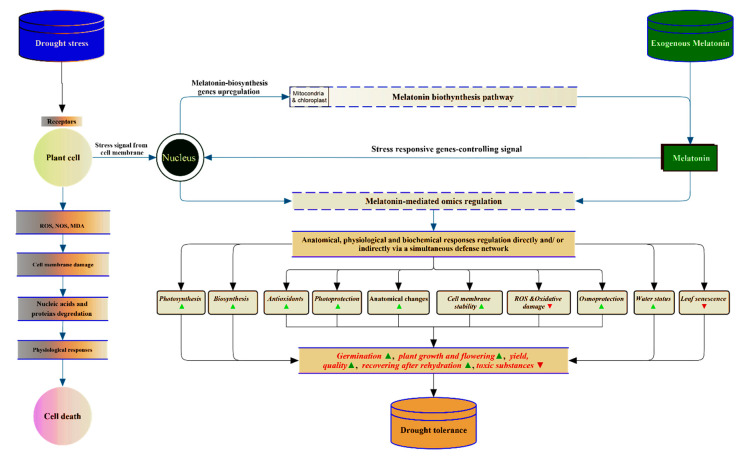
A schematic model explaining the mechanism underlying the melatonin-mediated drought stress response. At the cellular level, a stress signal from the cell membrane is received by the nucleus, which starts to activate the melatonin biosynthesis pathway from its precursor, tryptophan, in mitochondria and chloroplasts by upregulating the melatonin-biosynthesis genes. Melatonin sends its feedback on such stress to the nucleus to activate omics regulation. Consequently, the genes encoding the proteins related to plant anatomical, physiological, and biochemical responses are regulated directly and/or indirectly via a simultaneous defense network. The omics-mediated responses include photosynthesis, biosynthesis, enzymatic and nonenzymatic antioxidants, photoprotection, cell membrane stability, ROS and oxidative damage, osmoprotection, water status, and leaf senescence, in addition to the anatomical changes, which lead to drought tolerance. Consequently, the whole plant status is enhanced, including growth and development, flowering, yield, quality, and survival rate, while the toxic substances are decreased.

With the notable increase in global warming, rainfall disparity, and poor drainage, water stress (drought and waterlogging) is becoming one of the fiercest environmental challenges in the agriculture sector, mainly in the arid and semiarid regions for drought stress [1,2], and in the areas of heavy rainfall, inadequate draining, and flooding for waterlogging stress [3], which could seriously threaten food security by 2050, whenthe world’s population is predicted to reach ten billion [4]. The key impact of water stress is the massive generation of reactive oxygen species (ROS) and malondialdehyde (MDA) over the cell tolerance ability [5], therefore, directly and/or indirectly damaging the cell membrane, nucleic acids, and proteins (Figure 1). This adversely affects gas exchange and photosynthesis and decreases plant growth, as well as yield quality and quantity [6,7,8]. Practically, a global-scale analysis of published studies over the last four decades on maize and wheat revealed that 20–40% of yield reductions were due to water scarcity [9].Meanwhile, the destructive effect of waterlogging on crop yield has been estimated at a 40–80% loss in an area of more than 1.7 billion hectares [10,11,12].

Indeed, plants have developed several strategies to cope with water stress. In drought, plants avoid the drastic effects of stress through the induction of stomatal closure, accumulation of compatible solutes, and biosynthesis of wax [4]. Moreover, plants increase their tolerance by the activation of antioxidative abilities and the induction of some molecular chaperones to alleviate oxidative damage [8,13]. In waterlogging, plants avert stress by altering plant metabolism toward anaerobic, glycolytic, and fermentative metabolism. In response to anoxia, the plant activates the antioxidant machinery, expression of heat shock transcript, and accumulation of osmolytes [14]. Previous publications have stated that the various plant responses to water stress are mediated by essential regulators such as phytohormones [15]. Among them, melatonin is a unique antioxidant and plant master regulator that protects plants from oxidative stress and regulates various plant responses to environmental disorders, especially water stress [16,17,18]. Although accumulating reviews about the beneficial effects of melatonin have been published over the last decade, it still needs more discussion in order to update and discover melatonin functions, especially under biotic and abiotic stresses [19,20,21]. Herein, we will discuss the most recent and relevant studies of the protective roles of melatonin-induced water stress tolerance, including anatomical changes, and physiological and molecular mechanisms, as well as its central role in the hormonal system. Moreover, we will address the potential triple relationship, melatonin–nitric oxide–hydrogen sulfide, an emerging research point, in the light of previous water stress research. A grasp of the current situation and consideration of the future perspectives of the roles of melatonin in water stress tolerance will also be deeply discussed.

## 2. Melatonin-Induced Drought Stress Tolerance

### 2.1. An Overview

Among plant growth substances, melatonin (N-acetyl-5-methoxytryptamine) is an amazing and powerful naturally occurring antioxidant that effectively copes with the drastic effects of water deficit in plants [16,22]. Thus, melatonin is strongly recommended to mitigate drought stress in several plant species, including model plants [23,24], field crops [25,26], fruit crops [27,28], vegetable crops [29,30], as well as ornamental and medicinal plants [31,32] (Table 1). Melatonin treatment ranges from a very low concentration (50 nM) in grape [33] to a high dosage (1 mM) in maize [34] (Table 1). Moreover, melatonin can be applied in different forms to alleviate drought stress, including seed priming [35], seed coating [36], direct soil treatment [37], foliar application [32], in nutrient solutions and hydroponic systems [38], supplemented with irrigation [27], and roots pretreatment [39] (Table 1).

### 2.2. Melatonin is Involved in Drought Stress Tolerance

Given the wide use of melatonin in drought stress alleviation, it has been of interest for the scientific community to investigate the direct evidence of melatonin involvement in drought tolerance. This takes place through melatonin biosynthesis genes such as tryptophan decarboxylase (*TDC*), N-acetylserotonin methyltransferase (*ASMT*), serotonin N-acetyltransferase (*SNAT*), and caffeic acid O-methyltransferase (COMT). In this respect, the endogenous melatonin levels change with the alteration of the environmental conditions of plant growth. The melatonin level is increased, with a protective role, in response to different abiotic stressors such as cold, heat, heavy metals, UV radiation, water deficit, and waterlogging [18,22]. Thus, the expressions of the biosynthesis enzyme transcripts (*TDC*, *SNAT*, *ASMT*, and *COMT* genes) occur in stressful situations, producing a burst in the levels of endogenous melatonin. The global influence of environmental factors on the melatonin levels of plant organs was demonstrated in barley, tomato, and lupin plants by Arnao and coworkers [69,70,71]. Some representative examples of melatonin induction by drought can be consulted in studies on *Arabidopsis* [24], barley [49], bermudagrass [67], apple [53], grapevine [56], and rice [72]. In these cases, an increase in the melatonin level, between 2- and 6-fold, in one or more transcripts of melatonin biosynthesis enzymes due to stress conditions have been described [72,73].

### 2.3. Mechanisms of Melatonin-Induced Drought Stress Tolerance

#### 2.3.1. Anatomical Changes and Physiological Mechanisms

In the last few years, the role of melatonin as a multifunctional regulator of plant status under drought conditions, including (i) anatomical and (ii) physiological aspects, have been progressively studied and, notably, reached more than 42 studies within seven years (Table 1). (i) The anatomical changes are induced by melatonin within the different plant organs, including less cell membrane damage [63], more intact grana lamella of the chloroplast [45], alleviation of chloroplast ultrastructural damage and preservation of its system [33,61], safeguarding of the mitochondrial structure [67], maintenance of cell expansion [35], better leaf thickness, spongy tissue, and stomata size [33,35], cuticle formation [74], and wax accumulation [39]. (ii) By increasing drought severity, melatonin, which is biosynthesized in mitochondria and chloroplasts [75,76], exhibits more defense strategies. It promotes the physiological aspects, including the antioxidant system [27,59], to alleviate the oxidative damage, leading to less accumulation of reactive oxygen and nitrogen species (ROS and RNS) [25,52], less electrolyte leakage [41], lower lipid peroxidation (malondialdehyde reduction) [27,65], lower relative conductivity [57], the easing of toxic substances content [60], cellular redox disruption limitation [52], better nitro-oxidative homeostasis [52], and enhanced ascorbate (AsA)–glutathione(GSH) cycle capacity (higher GSH and AsA contents) [54]. Such beneficial effects are carried out by regulating enzymatic activity involving peroxidase (POD), ascorbate peroxidase (APX), catalase (CAT), and superoxide dismutase (SOD), as well as nonenzymatic antioxidants and osmoprotectants (proline and others) [37,44,64], and also secondary metabolites such as flavonoids, phenolics, and phenylalanine ammonialyase [48]. Simultaneously, melatonin improves the plant photosystem, as indicated by higher chlorophyll content [58], greater photosynthetic rates [43], and higher transpiration rates [31]. Moreover, melatonin has been proven to enhance photoprotection via improving photosystem II efficiency [34]. As a multifunctional substance, melatonin also regulates the osmotic potential of the cell [42] via the accumulation of soluble sugars and proline [62]. Moreover, water status is one of the most important priorities of melatonin to control under drought conditions. In this respect, melatonin enhances plant resistance via higher stomatal conductance [42], higher cell turgor and water holding capacity [65], and stomatal opening regulation [77]. Consequently, the whole plant status is enhanced, including seed germination efficiency [47], root generation vitality and strength [61], growth and flowering [36,50], visual quality [65], seed yield [38], leaf senescence alleviation [54], and quick recovery after rehydration [59].

#### 2.3.2. Molecular Mechanisms

##### Omics of Redox Hemostasis and Plant Built-In Processes

The protective mechanisms of melatonin have also been studied, and the ability of melatonin to protect plant cells against redox homeostasis disruption in response to drought stress has been focused on. Melatonin regulates ROS/reactive nitrogen species (ROS/RNS) levels and antioxidant-related genes, including SOD, POD, CAT, APX, glutathione S-transferase (*GSTP*), monodehydroascorbate reductase (*MDHAR*), dehydroascorbate reductase (*DHAR*), and glutathione reductase (*GR*) [27,30,32,37,43,45,52,56,59,78], as well as osmoprotective elements via the regulation of proline biosynthesis genes [52]. Melatonin not only alleviates oxidative damage, but also regulates plant built-in-associated genes, including carbohydrate/fatty and amino acids metabolism [26,36,37], the carbon metabolic pathway [67], nitrogen metabolism and transport [37,54], plant secondary metabolism [59], energy production [37,78], carotenoid metabolism and photosynthesis [27,36,37,59], and cuticle wax biosynthesis [74]. In this regard, the metabolism of carbohydrate/fatty acids has been reported to be upregulated via the seed-coating of soybean with a melatonin solution as a means to improve its tolerance to drought stress [36]. Melatonin is also a key regulator of nitrogen (N) metabolism and transport, as indicated by the higher expression levels of N uptake genes (*AMT2-1*, *AMT1-2*, *AMT1-6*, *AMT1-5*, *NRT1-1*, *NRT2-5*, *NRT2*, and *7NRT2-4*) and metabolic genes (*NADH-GOGAT NR*, *Fd-GOGAT*, *NiR*, and *GS*) in the leaves of apple trees [54].

##### Omics of Energy Production, Photosynthesis, and Wax Biosynthesis

Melatonin promotes energy production under water scarcity through regulating glycolytic protein expression and electron transport in the respiratory chain [78]. Moreover, melatonin governs the photosynthesis process via the regulation of molecular elements involved in the enzymatic activities of carbon dioxide (CO_2_)fixation (*PGK*, *TKT*, *FBA*, *RPI*, *FBP*, *GAPA*, *TIM*, *RPK*, *Rubisco*, *SEBP*, and *RPE*) [58], protein expression for carbon fixation [26], light reaction of photosynthesis (cytochrome P450) [37], and tetrapyrrole pigment biosynthesis [37,56,65]. Photosynthesis has also been reported to be upregulated via the seed-coating of soybean with a melatonin solution as a means to improve its tolerance to drought and salinity stress [36]. Among the interesting genes upregulated by melatonin, there are two subunits of photosystem I (*PS I*; *PsaG* and *PsaK*) and two elements (*PsbO* and *PsbP*) related to the oxygen-evolving complex of PS II (oxygen-evolving enhancer proteins) [36]. Moreover, melatonin upregulates the relative expression of the *PetF* ferredoxin gene(which controls the amount of reduced ascorbate and protects chlorophyll from degradation) and the *VTC4* gene, encoding the L-galactose 1-P-phosphatase for ascorbate biosynthesis [36]. In another study, Ma et al. [65] reported that melatonin inhibited the gene expression and enzyme activities of chlorophyll-degradation genes, including *chlase*, *Chl-PRX*, and *PPH*, in melatonin-treated plants during drought stress, which directly affects photosynthesis performance. On the other hand, Ding et al., [74] tested the relative expression of four wax biosynthesis-related genes, including *KCS1*(responsible for fatty acid elongation), *CER3*(involved in alkane synthesis), *TTS1*(associated with triterpenoids synthesis), and *LTP1*(accountable for lipids transport). It was remarked that the transcripts of the four genes were triggered by drought stress and were further induced as a result of melatonin treatment, demonstrating the role of melatonin in enhancing wax biosynthesis [74].

##### Omics of Stomatal Movement, Autophagy, and Others

Melatonin-mediated stomatal closure mechanism has also been investigated, suggesting that melatonin is a phytohormone that triggers stomatal closure via the signaling pathway ofPMTR1, which controls hydrogen peroxide (H_2_O_2_) production and the Ca^2+^ signalingtransduction cascade [77]. PMTR1 is a phytomelatonin receptor that has a receptor-like topology and interacts with the subunit of G-protein A (*GPA1*) in the plasma membrane [77]. The phytomelatonin–receptor binding drives the dissociation of Gγβ and Gα (heterotrimeric G-proteins), which triggers NADPH oxidase-dependent H_2_O_2_ release and activates Ca^2+^ as well as K^+^ efflux, leading to stomatal closure [77]. In addition, NAPDH oxidase, as a respiratory burst oxidase, generates superoxide radicals, which then undergoes dismutation to hydrogen peroxide either enzymatic or nonenzymatically. Under excessive drought, plants resort to getting rid of dysfunctional or unnecessary cellular components in order to facilitate the orderly degradation and recycling of cellular components through the autophagy mechanism. The regulatory role of melatonin in autophagy is elucidated in wheat seedlings via the enhancement of the metabolic process associated with autophagy, represented by the upregulation of the fused signal recognition particle receptor, Rab-related protein, serine protease, and aspartyl protease at the protein or mRNA level [78]. Moreover, melatonin regulates the action of key transcription factors such as *Myb4*, *AP37*, and zinc finger [41,67] in parallel with some transporter proteins, including proton transporter (*UCP1*), potassium transporter (*HKT1*), and water channel protein (*PIP2;1*) [41], which are all essential elements in stress tolerance. Moreover, melatonin application orchestrates some stress-signaling genes such as calcium and protein kinases-related genes, implying that kinase signaling could prove to have essential roles in drought tolerance [67].

All in all, as shown in Figure 1, it can be concluded that once the plants feel water scarcity under drought conditions, the protective and regulatory role of melatonin, in parallel with other anti-stress strategies, will start to prevent, alleviate, or stop the harmful effects of the stress [18,79]. At the cellular level, stress signals from the cell membrane inform the nucleus that “cell life is under threat” to cope with the drastic effects of the drought [77,80]. Quickly, the nucleus starts to activate the melatonin biosynthesis pathway from its precursor, tryptophan, in mitochondria and chloroplasts [75,76,81] through the upregulation of the melatonin-biosynthesis genes [53,65]. Remarkably, melatonin starts by sending its feedback on such stress to the nucleus to trigger the appropriate stress response through omics regulation [40,45,54,65]. As a result, the genes involved in the anatomical, physiological, and biochemical aspects are regulated directly and/or indirectly via a simultaneous defense network. The omics-mediated responses include photosynthesis, biosynthesis, antioxidants, photoprotection, cell membrane stability, osmoprotection, water status, leaf senescence, and oxidative damage alleviation, in addition to the anatomical changes. Consequently, the whole plant status is enhanced, including growth and development, flowering, yield, quality, and survival rate (recovering after rehydration), while the toxic substances are decreased, which collectively lead to drought tolerance.

#### 2.3.3. Melatonin Orchestrates other Phytohormones in the Regulatory–Defense Network

**Figure 2:**
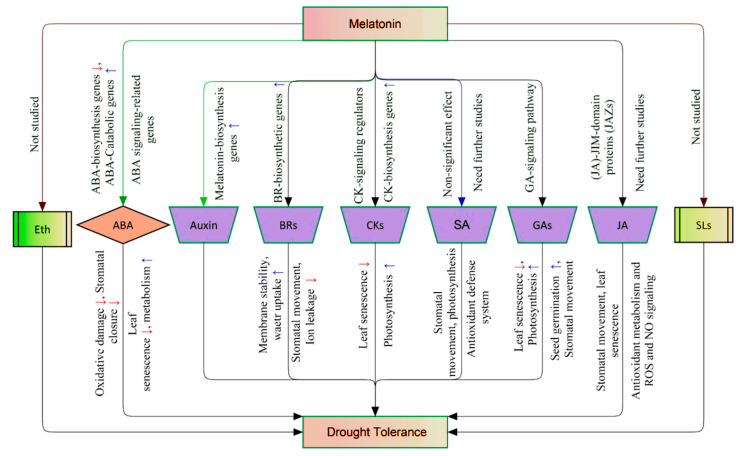
A schematic model explaining the effect of melatonin on other phytohormones under drought stress: Under drought, melatonin enhances the levels of brassinosteroids (BRs), cytokinins (CKs), gibberellins (GAs), and jasmonates (JAs) and decreases the abscisic acid (ABA) level and auxins. Eth, ethylene; ABA, abscisic acid; BRs, brassinosteroids; CKs, cytokinins; SA, salicylic acid; GAs, gibberellins; JA, jasmonic acid; SLs, strigolactones. Red connectors, not studied; green connectors, reduced; black connectors, enhanced; blue connectors, nonsignificant effect. **↑**, upregulated; **↓**, downregulated.

Melatonin is a central molecule in the hormonal system and, thus, increases plant tolerance to drought stress through the regulation of phytohormone levels such as abscisic acid (ABA), auxins (Auxs), cytokinins (CKs), gibberellins (GAs), brassinosteroids (BRs), jasmonic acid (JA), and salicylic acid (SA). The key physiological aspects that are much regulated by phytohormones in response to drought stress include antioxidant metabolism, carbohydrate production (carbon metabolism), stomatal movement, and leaf senescence [82]. Drought stress upregulates ABA, BRs, and JA [59,82] and downregulates CKs and GAs [51,59], while melatonin enhances the levels of BRs, GAs, JA, and CKs [59] and decreases the ABA level [59] (Figure 2).

Water scarcity stimulates abscisic acid (ABA) biosynthesis [13,83], which in turn downregulates the main metabolic pathways [59], induces stomatal closure [82], and contributes to leaf senescence [82]. Moreover, the overaccumulation of abscisic acid upregulates the ROS generation pathways and causes oxidative damage [13]. However, melatonin maintains the abscisic acid homeostasis (low to moderate concentrations) by positive regulation of its biosynthetic genes and negative regulation of the catabolic genes [49,51,53,59]. Li et al. [53] clarified that melatonin effectively downregulates *MdNCED3*, an abscisic acid synthesis gene, and upregulates its catabolic genes, *MdCYP707A2* and *MdCYP707A1*, causing abscisic acid reduction. Moreover, melatonin regulates abscisic acid signaling-related genes such as *SnRK2* (SNF1-related protein kinases 2), *RCAR*/*PYR*/*PYL*, and *NCED* (nine-cis-epoxycarotenoid dioxygenase) [67]. Cytokinins (CKs) are an essential group of phytohormones in the inhibition of leaf senescence and chlorophyll degradation under water stress, which in turn suppresses cytokinin biosynthesis and transport, causing cytokinin reduction and faster leaf senescence [84,85,86]. Melatonin treatment upregulates cytokininlevels and some related signaling factors, leading to better photosynthesis efficiency and drought-induced tolerance [59,65,87]. The first demonstration that melatonin inhibits leaf senescence was made in barley [88]. Melatonin-induced alleviation of leaf senescence in creeping bentgrass is associated with the downregulation of chlorophyll catabolism and synergistic interaction with cytokinins-biosynthesis genes and signaling pathways in melatonin-treated ipt-transgenic plants [65].

Brassinosteroids (BRs) possess an apparent ability as drought stress-protective molecules in plants [89]. Melatonin regulates the biosynthesis of brassinosteroidsvia the stimulation of various brassinosteroid–biosynthetic genes like DWARF4, D11, and RAVL1 [90], which control stomatal movement [91], enhance cell membrane constancy and water uptake, and decrease membrane damage-induced ion leakage in the case of water limitation [59,92]. Jasmonic acid (JA) is a crucial plant hormone in the regulation of drought responses such as stomatal movement, leaf senescence, antioxidant metabolism, and ROS and nitro-oxide signaling [93,94,95,96,97,98]. Jasmonic acid levels are increased in response to drought stress and are highly stimulated as a result of melatonin application, which induces drought tolerance [59]. The melatonin–jasmonic acid crosstalk is stated by regulating molecular transcripts such as JA–JIM-domain proteins (JAZs) in jasmonic acid signaling [67].

Moreover, melatonin interacts with gibberellins (GAs) via GA-signaling, which further controls the biosynthesis of auxins [24,59]. Gibberellins are regulators of stomatal movement [99,100], photosynthesis [101], seed germination [102], and leaf senescence [4]. Drought stress inhibits gibberellin biosynthesis [51,103], which is much enhanced in response to melatonin treatment, causing drought tolerance [59]. Salicylic acid (SA) accumulation plays a vital role in stomatal movement, photosynthesis, and the antioxidant defense system [4]. In maize plants, under drought conditions, an increase (but nonsignificant) in the defense hormonesalicylic acid has been described in melatonin-treated plants [34]. Enhanced drought tolerance was achieved using mainly transgenic plants through the overexpression of melatonin-biosynthesis genes under drought conditions [24,104,105], which led to a decrease of indole-3-acetic acid (IAA) that may be due to the competition for the same precursor, tryptophan. The plant root is the first plant organ to touch the environment, and it represents a priority for plant breeders to improve its efficiency under abiotic stresses, including drought. Interestingly, melatonin targets plant roots, showing an auxin-like action [106]. In this regard, Pelagio-Flores et al. [106] provided direct evidence supporting the mechanism of this action in *Arabidopsis thaliana* via inspiring lateral and adventitious root formation, conferring a widespread root system. The auxin-like effect of melatonin in roots was elucidated using auxin-responsive marker constructs. It was suggested that melatonin neither activates auxin-inducible gene expression nor induces the degradation of *HS:AXR3NT-GUS*, indicating that root developmental changes elicited by melatonin are independent of auxin signaling [106]. To date, under drought situations, there has been no comprehensive study revealing the interaction between melatonin and ethylene or strigolactones; thus, further investigations are needed. All the above details confirmed that melatonin acts as a relevant regulator of many plant hormone elements, a so-called plant master regulator [107,108], making the plants more tolerant when irrigation water is limited (Figure 2).

#### 2.3.4. The Crosstalk of Melatonin, Nitric Oxide, and Hydrogen Sulfide in Melatonin–Water Stress Research

Melatonin, nitric oxide (NO), and hydrogen sulfide (H_2_S) are essential small molecules in the plant defense network [109]. Melatonin controls various plant responses under water stress, as described throughout the text. Nitric oxide is a fundamental signaling molecule working as a pro-oxidant and antioxidant element against adverse environments, which is determinant by its endogenous concentration and locational production status [110]. Hydrogen sulfide is a master metabolic regulator in plants, which alleviates the destructive effects of environmental stresses such as drought and waterlogging through the regulation of enzymatic antioxidants [111,112]. The relationship of melatonin, nitric oxide, and hydrogen sulfide has been studied in fruit ripening regulation [113], as well as under biotic [114] and abiotic stresses [110] such as salinity [115] and drought [52]. For instance, nitric oxide and ethylene crosstalk is mediated by hydrogen sulfide and melatonin activity, which regulate various metabolic pathways associated with fruit ripening [113]. Moreover, salt stress alone or combined with iron deficiency expands endogenous hydrogen sulfide and nitric oxide, which are much enhanced due to melatonin treatment [116]. To date, there has only been one published report addressing the relationship between melatonin and nitric oxide under water scarcity [52], while the melatonin–hydrogen sulfide relationship and the triple crosstalk of melatonin–nitric oxide–hydrogen sulfide under water stress remain unknown. In that report, the authors suggested that melatonin mitigates drought damage in alfalfa plants by modulating nitro-oxidative homeostasis through the regulation of reactive oxygen and nitrogen species metabolic enzymes at the enzymatic and/or transcript level [52]. However, how endogenous melatonin interacts with nitric oxide under water scarcity is still a research point [110]. The question that still needs to be answered is whether the crosstalk of melatonin, nitric oxide, and hydrogen sulfide under water stress is similar to the situation under other environmental stresses or if they have a unique interaction in each situation.

## 3. Melatonin-Induced Waterlogging Stress Tolerance

### 3.1. An Overview

Despite the importance of melatonin in mitigating the harmful effects of abiotic stresses, the research on melatonin-induced waterlogging tolerance has only recently started to emerge (Table 2). The first report was registered as a patent in 2015 by Chen et al. [117]. In this report, the authors indicated that melatonin has a great ability to eliminate ROS, alleviate oxidative damage, resist waterlogging, and, consequently, revert losses in yield and quality [117]. After this ground-breaking work, Zheng et al. [118] elucidated that melatonin is an effective phytohormone to protect apple plants under waterlogging stress. Melatonin application improved endogenous melatonin levels, antioxidant enzyme activities, chlorophyll content and photosynthesis, and aerobic respiration, while it suppressed chlorosis, wilting, ROS, malondialdehyde, and anaerobic respiration [118]. Moreover, melatonin-biosynthesis enzymes (*MbT5H1*, *MbAANAT3*, and *MbASMT9*) were upregulated due to melatonin treatment [118]. In recent work, Zhang et al. [119] investigated the impact of melatonin pretreatment on alfalfa under waterlogging stress and indicated that melatonin could alleviate the stress damage and enhance plant growth, chlorophyll content, and PSII efficiency. Moreover, melatonin treatment increased polyamine (putrescine, spermidine, and spermine) levels and decreased ethylene under stress, which are controlled via changes in gene expression [119].

### 3.2. Mechanisms of Melatonin-Mediated Waterlogging Stress Tolerance

**Figure 3:**
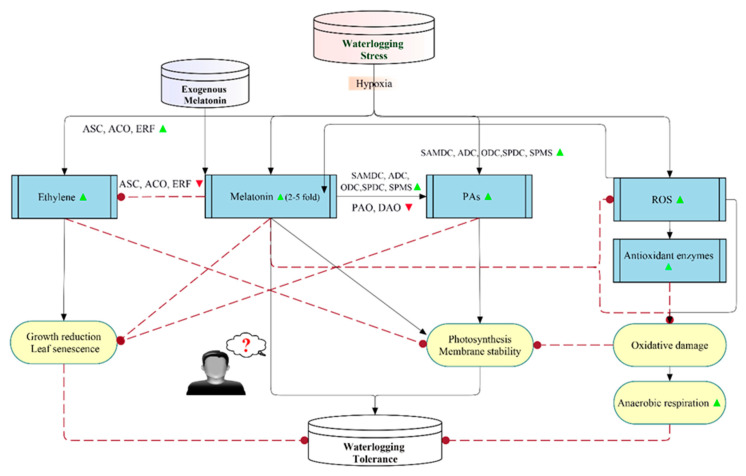
Schematic model explaining the protective mechanisms of melatonin in waterlogging tolerance. The solid arrows indicate stimulation, while the dashes indicate inhibition. ▲ and ▼ shapes indicate enhanced or decreased levels, respectively. Waterlogging induces ethylene, melatonin (2- to 5-fold), polyamines (PAs), and reactive oxygen species (ROS). Melatonin is also induced in response to ROS generation and exogenous melatonin. Melatonin stimulates PA biosynthesis, photosynthesis, and membrane stability, while it inhibits ethylene biosynthesis, growth reduction, leaf senescence, ROS, and oxidative damage. Excessive ROS causes oxidative damage leading to anaerobic respiration, which is scavenged by antioxidant enzymes. Additionally, growth reduction and leaf senescence are increased by ethylene, while they are decreased by PAs. Moreover, photosynthesis and membrane stability is enhanced by PAs, while they are reduced by ethylene induction and oxidative damage. The role of melatonin in waterlogging tolerance still needs further study. This figure is a combination of the two published mechanisms of Zheng et al. [118] and Zhang et al. [119], with some modifications.

Melatonin application is a practical approach to suppress the drastic effects of waterlogging (Figure 3). To date, there are two published mechanisms induced by melatonin to enforce waterlogging tolerance [118,119]. Zheng et al. [118] proposed the first mechanism of melatonin-mediated waterlogging tolerance in apple seedlings, which keeps aerobic respiration and preserves photosynthesis by efficient repression of the ROS burst and consequent mitochondrial degradation. Zhang et al. [119] suggested another model in alfalfa through interaction with or direct regulation of the metabolic pathways of ethylene and polyamines (PAs). Waterlogging stress induced an increase of endogenous melatonin levels of 2- to 5-fold compared with unstressed plants. Melatonin starts by reducing ethylene production via the downregulation of ethylene synthesis-associated genes and alleviation of waterlogging-caused growth inhibition, chlorosis, and premature senescence [119]. Then, melatonin enhances polyamines levels by promoting the gene expression of the involved enzymes in polyamine metabolism [119]. The authors proposed that melatonin increases waterlogging tolerance, at least partially, by regulating polyamines and ethylene biosynthesis due to ethylene suppression and polyamine promotion, leading to more stable cell membranes, better photosynthesis, and less ethylene-responsive senescence [119].

Collectively, waterlogging induces ethylene, melatonin (2- to 5-fold), polyamines (PAs), and ROS. Melatonin is also produced in response to ROS generation and exogenous melatonin. Melatonin stimulates polyamines biosynthesis, photosynthesis, and membrane stability, while it inhibits ethylene biosynthesis, growth reduction, leaf senescence, ROS, and oxidative damage. Excessive ROS causes oxidative damage leading to anaerobic respiration, which is scavenged by antioxidant enzymes. Additionally, growth reduction and leaf senescence are increased by ethylene, while they are decreased by polyamines. Moreover, photosynthesis and membrane stability are enhanced by polyamines, while they are reduced by ethylene induction and oxidative damage (Figure 3). The role of melatonin in waterlogging tolerance, especially molecular evidence, still needs further study.

## 4. Conclusions

Water stress tolerance (drought stress and waterlogging) may be attributed to structural and functional adaptations at the cellular and whole-plant levels, including root enhancement, growth promotion, oxidative damage alleviation, osmotic potential modulation, leaf water potential, cell wall elasticity control, stomatal closure, and the accumulation of osmolytes, thereby easing the harmful impacts of such destructive stresses [4,14]. Melatonin may be considered a core part of the regulatory network controlling all of these mechanisms, and it represents a promising material for future studies and practical use [18,107,108]. Melatonin research has been experiencing hypergrowth in the last two decades; however, its roles in water stress tolerance need further investigation. The regulation of melatonin and its metabolism pathway under water stress is still unclear. Understanding the role of melatonin in nutrient uptake will give us an excellent opportunity to take advantage of such a useful molecule for strengthening plant tolerance and adaptability to water stress. Furthermore, in-depth studies to clarify the molecular mechanisms using microarray, transcriptomic, and proteomic analyses will help to figure out the genes regulating plant anatomical, physiological, and biochemical aspects in response to exogenous melatonin applications under water stress. Exploring new receptor-mediated phytomelatonin signaling plays a role in such physiological processes in future works. Additionally, the best-known information on the relationship of melatonin with other small signaling molecules, such as NO and H_2_S, can be relevant. In recent decades, significant advancement in the knowledge of the mechanism of NO and H_2_S signaling and their crosstalk with melatonin has been made [113,115]. Therefore, the distribution of melatonin in plant organs and their interrelations with NO and H_2_S should be further studied [115]. The molecular mechanisms revealing the crosstalk between melatonin and other phytohormones such as strigolactones and ethylene in promoting water stress tolerance are worth further studies on mutagenesis or genetic modulation and aquatic model plants. The relationship between melatonin and multiple stressor combinations is a topic to be taken into account in future research due to the complexity of the interaction of plants with diverse environmental agents. Lastly, the use of synthetic melatonin, a relatively cheap compound, or phytomelatonin-rich extracts should be an interesting approach to improving plant tolerance [120,121].

## Figures and Tables

**Table 1:** Roles of melatonin in drought stress tolerance.

Common Name	Scientific Name	Drought Treatment	Melatonin Treatment		Effects	Reference
			Concentration*	Application Form		
Model Plants						
Arabidopsis	*Arabidopsis thaliana*	Water withholding (21 d)	50 µM	Supplemented with nutrient solution	Stress-responsive genes ▲, soluble sugars ▲	[40]
**Field Crops**						
Rice	*Oryza sativa*	Water drainage from vessels (5 d)	100 μM	Pretreatment in growing distilled water	Plant growth ▲, osmoprotectants proline ▲, stress-responsive genes ▲, mitochondrial structure ▲, ROS ▼, electroleakage ▼	[41]
Maize	*Zea mays*	Water withholding (8 d), melatonin application during recovery, followed by withholding (8 d).	1 mM	Supplemented with irrigation	Photoprotection (PSII efficiency) ▲	[34]
Maize	*Z. mays*	30–60% SWC (8d)	100 µM	Foliar application	Recovering after rehydration ▲, photosynthesis ▲, stomatal conductance ▲, transpiration rates ▲, cell turgor and water holding capacity ▲, enzymatic and nonenzymatic antioxidants ▲, osmotic potential ▼, ROS ▼	[42]
Maize	*Z. mays*	20% PEG6000(3 d)	10–100 μM	Foliar application pre-treatment	Photosynthesis ▲, antioxidant enzymes ▲, carbon fixation ▲, amino acids and secondary metabolites biosynthesis ▲, ROS ▼	[26]
Maize	*Z. mays*	Water withholding (7 d)	100 µM	Two methods (root-irrigation and foliar application)	Photosynthesis ▲, ROS ▼	[43]
Maize	*Z. mays*	40–45% field capacity (50 d)	50 µM (foliar spray) and 100 µM (soil drench)	Foliar application or soil treatment	Photosynthesis ▲, antioxidant enzymes ▲, ROS ▼	[44]
Wheat	*Triticum aestivum*	40% and 60% field capacity (7 d)	500 µM	Soil application	Chloroplast structure▲, photosynthesis ▲, cell turgor and water holding capacity ▲, GSH and AsA contents ▲, antioxidant enzymes▲, GSH–AsA cycle-related genes ▲, ROS ▼, membrane damage ▼	[45]
Wheat	*T. aestivum*	30% pot holding capacity (8 d)	100 µM	Soil application	Recovering after rehydration ▲, biomass and root/shoot ratio ▲, water holding capacity ▲, chlorophyll ▲, photosynthesis ▲, ROS ▼, MDA ▼	[46]
Wheat	*T. aestivum*	20% PEG 6000 (7 d)	10 and 100 μM (variety-dependent)	Seeds treatment	Germination percentage ▲, germination index ▲, germination potential ▲, radicle length and number ▲, plumule length ▲, lysine (germination-related amino acid) ▲	[47]
Tartary Buckwheat	*Fagopyrum tataricum*	20% field capacity (15 d)	100 μM	Foliar application	Water status ▲, osmoprotection ▲, secondary metabolites▲, antioxidant enzymes▲, photosynthesis ▲, ROS ▼	[48]
Barley	*Hordeum vulgare*	(Combined drought and cold)	1 mM	Foliar or soil application	Endogenous melatonin▲, ABA ▲, water status ▲, antioxidants ▲, photosynthesis ▲, PSII efficiency ▲	[49]
Soybean	*Glycine max*	20% field capacity (10 d)	50 µM	Seed coating	Seedlings growth ▲, biomass ▲, electrolyte leakage ▼	[36]
Soybean	*G. max*	15% PEG 6000 (7 d)	100 µM	Supplemented with nutrient solution	Seedlings growth ▲, photosynthesis ▲	[38]
Soybean	*G. max*	45% RSWC (15 d)	100 µM	Foliar application	Antioxidant enzymes ▲, osmolytes ▲, MDA ▼	[25]
Soybean	*G. max*	15% PEG6000 (3 d)	100 μM	Foliar and root application	Plant growth and flowering ▲, seed yield ▲, gas exchange▲, PSII efficiency ▲, antioxidant enzymes ▲, MDA ▼	[50]
Cassava	*Manihot esculenta*	20% PEG 6000 (11 d)	100 µM	Soil application	POD activity▲, ROS ▼	[37]
Cotton	*Gossypium hirsutum*	10% PEG 6000 (7 d)	100 µM	Seeds pre-soaking	Number and opening of stomata in cotton testa ▲, germination parameters▲, antioxidant enzymes ▲, osmoprotection ▲, GA3 ▲, ABA ▼, ROS ▼, MDA ▼	[51]
Alfalfa	*Medicago sativa*	Water withholding (7 d)	10 µM	Soil application	Chlorophyll ▲, stomatal conductance ▲, osmoprotection ▲, Nitro-oxidative homeostasis ▲, cellular redox disruption ▼,MDA ▼, ROS ▼	[52]
**Fruits**						
Apple	*Malus* spp.	Water withholding (6 d)	100 µM	Soil application	Water holding capacity ▲, chlorophyll ▲, photosynthesis ▲, antioxidants ▲, stomatal opening regulation ▲, melatonin biosynthesis genes ▲, electrolyte leakage ▼, ROS▼, ABA ▼ through ABA synthesis gene▼ and catabolic genes ▲	[53]
Apple	*M. domestica*	50% field capacity (3 months with sampling every month)	100 µM	Soil application	Plant growth ▲, nutrients uptake fluxes ▲, N metabolism ▲, endogenous melatonin ▲, chlorophyll ▲, photosynthesis ▲, relative water content ▲, stomatal status ▲, electrolyte leakage ▼, ROS ▼	[54]
Apple	*M. domestica*	50% field capacity (3 months with sampling every month)	100 µM	Soil application	Chlorophyll ▲, photosynthesis ▲, photoprotection ▲, antioxidant enzymes ▲, GSH and AsA contents ▲, oxidative damage ▼, leaf senescence ▼, senescence-associated gene 12 ▼, pheophorbide a oxygenase-related gene ▼, ROS▼	[55]
Grape	*Vitis vinifer*	10% PEG 6000 (12 d)	50, 100 and 200 nM	Roots pretreatment	Photoprotection ▲, leaf thickness ▲, spongy tissue ▲, stoma size ▲, chloroplast structure ▲, enzymatic and nonenzymatic antioxidants ▲, osmoprotectants (free proline) ▲, ultrastructural damage ▼, oxidative injury ▼	[33]
Grapevine	*V. amurensis V. vinifera* and *V. labruscana*	10% PEG 6000 (4 d)	Endophyte colonization of secreted-melatonin bacteria	*Bacillus amyloliquefaciens* SB-9 colonization	Melatonin synthesis and its intermediates ▲, plant growth ▲, ROS ▼, MDA ▼	[56]
Grape	*V. vinifer*	Water withholding (18 d)	100 μM	Supplemented with irrigation	MDA ▼, relative conductivity ▼	[57]
Grape	*V. vinifer*	Water withholding (18 d)	100 μM	Supplemented with irrigation	Chlorophyll ▲, SOD activity ▲	[28]
Kiwifruit	*Actinidia. chinensis var. deliciosa*	Water withholding (9 d) (RWC below 35% field capacity)	100 μM	Supplemented with irrigation	Root vigor ▲, osmoprotectants ▲, proteins biosynthesis ▲, chlorophyll ▲, photosynthesis ▲, light energy absorption ▲, photoprotection ▲, CO_2_ fixation-associated genes ▲, MDA ▼, cell membranes damage ▼, stomatal closure ▼	[58]
Kiwifruit	*A. chinesis*	water withholding (9 days)	100 µM	Irrigation pretreatment	Water holding capacity ▲, antioxidant enzymes-related genes ▲, GSH–AsA cycle-related genes ▲, ROS ▼, MDA ▼	[27]
Chinese hickory	*Carya cathayensis*	30% PEG 6000 (10–40 d)	100 µM	Foliar application pretreatment	Recovering after rehydration ▲, photosynthesis ▲, antioxidants ▲, osmoprotectants ▲, metabolic pathways-related genes ▲, antioxidant enzymes-related genes ▲, ROS ▼	[59]
**Vegetables**						
Tomato	*Solanum lycopersicum*	Water withholding for (5–20 d after moderate drought)	0.1 mM	Supplemented with irrigation	Photosynthesis ▲, root vigor ▲, PSII efficiency ▲, antioxidants ▲, toxic substances ▼	[60]
Tomato	*S. lycopersicum*	10% PEG (7 d)	200 µM	Foliar application	Chlorophyll ▲, p-coumaric acid content ▲, antioxidant enzymes ▲, MDA ▼	[29]
Pepper	*Capsicum annuum*	10% PEG (8 d)	50 µM	Seed pretreatment	Water holding capacity ▲, endogenous melatonin ▲, GSH content ▲, chlorophyll ▲, carotenoids ▲, proline ▲, antioxidant enzymes ▲, MDA ▼	[30]
Watermelon	*Citrullus lanatus*	Water withholding (4 d)	150 µM	Root pretreatment	Wax accumulation ▲, melatonin–ABA crosstalk ▲	[39]
Cucumber	*Cucumis sativus*	18% PEG 6000 (days)	100 µM	Seeds priming and nutrient solution	Seed germination ▲, root growth ▲, root/shoot ratio ▲, roots vigor ▲, chlorophyll ▲, photosynthesis ▲, chloroplasts ultrastructure ▲, antioxidant enzymes ▲, ROS ▼	[61]
Rapeseed	*Brassica napus*	4% PEG 6000 (7 d)	0.05 mM	In PEG solution	Plant growth ▲, antioxidants ▲, osmoprotectants ▲, ROS ▼	[62]
Rapeseed	*B. napus*	−0.3 and −0.4 Mpa PEG 6000 (7 d)	500 µM	Seed priming	Chlorophyll ▲, stomatal regulation ▲, chloroplast structure ▲, cell expansion and cell wall ▲, antioxidant enzymes ▲, osmoprotectants ▲, oxidative injury ▼	[35]
**Ornamental and Medicinal Plants**						
Jinyu Chuju	*Dendranthma morifolium*	40% field capacity (6 d)	100 µM	Foliar application	Chlorophyll ▲, photosynthesis ▲, biomass ▲, osmoprotectants (TSS and proline) ▲, cell membrane damage ▼, relative conductivity ▼, MDA ▼	[63]
Moldavian balm (Dragon head)	*Dracocephalum moldavica*	40–60% field capacity	100 µM	Foliar application	Plant growth and flowering ▲, antioxidants ▲, chlorophyll ▲, water holding capacity ▲, ROS ▼, MDA ▼	[64]
Creeping bentgrass	*Agrostisstolonifera*	Water withholding (14 d)	20 μM	Foliar application	Visual quality ▲, PSII efficiency ▲, chlorophyll ▲, water holding capacity ▲, melatonin biosynthesis genes ▲, dehydration responsive genes ▲, Chlorophyll-degradation genes ▼, leaf senescence ▼, ROS ▼, MDA ▼	[65]
Tall fescue	*Festuca arundinacea*	Water withholding (10 d)	20 μM	Irrigation pretreatment	Plant growth ▲, chlorophyll ▲, antioxidant enzymes ▲, ROS ▼, MDA ▼	[66]
Bermudagrass	*Cynodon dactylon*	Withholding water (21 d)	20 and 100 μM	Irrigation pretreatment	Plant growth ▲, chlorophyll ▲, survival rate ▲, antioxidant enzymes ▲, stress-responsive genes ▲, metabolic regulation ▲, hormonal signaling-related genes regulation ▲, ROS ▼	[67]
Fenugreek	*Trigonella foenum-graecum*	19.5% PEG 6000(21 d)	100 and 300 μM	Foliar application pre-treatment	Endogenous melatonin and secondary metabolites ▲, chlorophyll ▲, antioxidant enzymes ▲, ROS ▼	[68]
Coffee	*Coffea arabica*	40% of max moisture retention capacity (21 d)	300 μM	Soil application	Root vigor ▲, photoprotection ▲, gas exchange ▲, carboxylation efficiency ▲, chlorophyll ▲, antioxidants ▲, MDA ▼	[31]
Tea	*Camellia sinensis*	20% PEG 6000 (2 d)	100 µM	Foliar application pre-treatment	Photosynthesis ▲, GSH and AsA contents ▲, antioxidant enzymes ▲, antioxidant enzymes-related genes ▲, ROS ▼, MDA ▼	[32]
**Other Crops**						
Tobacco, Tomato and Cucumber	*Nicotiana benthamiana, S. lycopersicum* and *C. sativus*	Water withholding (6 d)	10 μM	Foliar application	MDA ▼, drought tolerance ▲	[23]

▲ or ▼, enhanced or decreased compared to control. ROS, reactive oxygen species; PSII, photosystem II; GSH, glutathione; AsA, ascorbate; MDA, malondialdehyde; ABA, abscisic acid; GA3, gibberellic acid; SOD, superoxide dismutase; POD, peroxidase; TSS, total soluble sugar. * Only those doses of exogenous melatonin that had a superior positive impact on plant tolerance against drought stress have been selected.

**Table 2:** Roles of melatonin in waterlogging stress tolerance.

Species	Scientific Name	Waterlogging Treatment	Melatonin Treatment		Functions	References
			Concentration *	Application Form		
Apple	*Malus baccata*	Waterlogging stress (9 d)	200 µM (foliar spraying) 600 µM (root irrigation)	Foliar spraying or root irrigation	Endogenous melatonin ▲, antioxidant enzymes ▲, chlorophyll ▲, photosynthesis ▲, aerobic respiration ▲, synthetic enzymes ▲ ROS ▼, MDA ▼, anaerobic respiration ▼, chlorosis and wilting ▼	[118]
Alfalfa	*Medicago sativa*	Waterlogging stress (10 d)	100 µM	Foliar spraying pretreatment	Endogenous melatonin ▲, gene expression regulation ▲, photosynthesis ▲, electroleakage ▼, MDA ▼, leaf senescence ▼, polyamine and ethylene metabolism reprogramming	[119]

▲ or▼, enhanced or decreased compared to control. ROS, reactive oxygen species; MDA, malondialdehyde. * Only those doses of exogenous melatonin that had a superior positive impact on plant tolerance against waterlogging stress have been selected.
